# Unlocking Zn‐Ion Diffusion in Disordered Rocksalt Cathodes for Nonaqueous Zn‐Ion Batteries

**DOI:** 10.1002/anie.8007340

**Published:** 2026-07-13

**Authors:** Zixuan Li, Rui Qi, Yee Chit Wong, Longlong Wang, Jun Chen, Yi Yuan, Reinhard J. Maurer, Robert A. House, Nicholas D. M. Hine, Peter G. Bruce, Alex W. Robertson

**Affiliations:** ^1^ Department of Materials University of Oxford Oxford UK; ^2^ Department of Physics University of Warwick Coventry UK; ^3^ Department of Chemistry University of Warwick Coventry UK; ^4^ University of Vienna Faculty of Physics Vienna Austria; ^5^ Department of Chemistry University of Oxford Oxford UK

**Keywords:** cation vacancy engineering, disordered rocksalt cathodes, multivalent‐ion transport, nonaqueous electrolytes, nonaqueous zinc‐ion batteries, oxygen redox, Zn^2^^+^ intercalation

## Abstract

Multivalent batteries, particularly zinc‐ion batteries (ZIBs), are promising candidates for high‐energy–density energy storage. However, their development is hindered by a scarcity of suitable cathode materials capable of the reversible (de)intercalation of Zn^2+^. To address this challenge, we propose cation‐disordered rocksalt (DRX) cathodes, which have demonstrated excellent performance in Li‐ion batteries, as a versatile host framework for nonaqueous ZIBs. Specifically, a vacancy‐containing Mn_0.4_Ti_0.4_O_2_ DRX cathode demonstrates a reversible capacity of 170 mAh g^−1^ in nonaqueous ZIBs. Our investigation reveals that the Zn^2+^ ionic diffusion mechanism within the DRX framework is intrinsically sluggish compared to monovalent ions like Li^+^ due to strong electrostatic repulsion. Therefore, to successfully unlock Zn^2+^ migration, we show that it is necessary to introduce cation vacancies into the host, which significantly reduces the ion migration barrier. Additionally, we suggest that anion engineering may further enhance diffusion kinetics. This work expands the cathode material landscape for ZIBs and provides general insights into the design of disordered hosts for multivalent ion storage.

## Graphical abstract

Developing viable cathode materials for multivalent battery chemistries is challenging due to the intrinsic sluggish diffusion kinetics. A disordered rocksalt oxide host was found to “lock” the cycling of zinc ions from nonaqueous electrolyte. We unlock zinc ion diffusion in the disordered rocksalt by introduction of a percolating vacancy network.

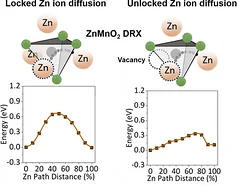

## 1 Introduction

The growing global demand for energy storage has driven interest in sustainable and low‐cost alternatives to lithium‐ion batteries. Multivalent battery systems based on ions such as Zn^2+^, Mg^2+^, Ca^2+^, and Al^3+^ are particularly attractive due to their high theoretical energy density arising from multi‐electron transfer [1, 2]. Among these systems, Zn‐based battery chemistries have emerged as one of the most extensively studied candidates because of the versatile electrochemistry options on the cathode side and their compatibility with both aqueous and nonaqueous electrolytes [3, 4]. While aqueous Zn‐ion batteries (ZIBs) have seen rapid progress, and come with significant advantages, concerns remain over their viability due to Zn corrosion, hydrogen evolution, and the limited electrochemical stability window of water [5]. Harnessing the advantages of nonaqueous Zn electrolytes instead, particularly their wider voltage window and a more stable electrode–electrolyte interface, may offer a more viable route toward a practical multivalent rechargeable battery.

Despite this promise, in nonaqueous multivalent batteries, including Zn‐based systems, a major challenge on the cathode side is the sluggish diffusion of ions in solid materials, which severely limits the range of viable intercalation‐type materials [6, 7]. The high charge density of multivalent ions results in strong electrostatic interactions between cations and host anion lattices (compared to monovalent Li^+^ and Na^+^), substantially reducing ionic mobility [6]. Although the reported intercalation hosts for Zn^2+^ are more than those of other multivalent ions, [2] the number of viable cathode materials remains limited. Reported examples include spinel oxides such as ZnAl_x_Co_2‐x_O_4_ [8], layered oxides such as V_2_O_5_ [9] and δ‐MnO_2_ [10], layered sulfides such as VS_2_ [11] and TiS_2_ [12], and Prussian blue analogs such as KNiHCF [13]. However, many of these cathodes rely on costly or toxic elements such as Co, Ni, or V, while sulfide‐based cathodes generally suffer from low operating voltages. Expanding this limited repertoire of candidate intercalation‐type oxide cathodes is therefore desirable, as it could theoretically offer high voltage transition metal redox across a broader chemical space while being compatible with nonaqueous electrolytes. New cathode materials that are more cost‐effective, supply chain secure, and nontoxic would also be attractive.

One promising class of materials for advanced cathodes is the disordered rocksalt (DRX) family, which has demonstrated exceptional performance in Li‐ion batteries, particularly in Li‐rich compositions [14]. In the crystal structure of DRX materials, an interconnected network of tetrahedra without transition metal site occupancy provides a low energy pathway for Li^+^ diffusion. These 0‐transition‐metal (0‐TM) channels form a percolating network for long‐range ion transport [15]. Combined with their high capacity and remarkable compositional flexibility, these features observed in Li‐based DRX cathodes make the Zn‐based DRX materials attractive candidate host structures for ZIBs. We have previously reported these Zn based DRX materials, including ZnMnO_2_, and have evaluated their performance for aqueous ZIBs, but multi‐modal characterization revealed that Zn^2+^ could not be deintercalated from the DRX lattice [16]. Instead, the structure underwent an irreversible transformation to a spinel‐like Zn_x_Mn_y_O_3_ phase during the first charge, with subsequent charge storage dominated by Mn dissolution and redeposition rather than intercalation [16]. Whether facile Zn^2+^ diffusion can be realized in Zn‐based DRX materials in nonaqueous electrolytes remains an open question.

In this work, we were able to successfully unlock Zn^2+^ diffusion in Mn_0.4_Ti_0.4_O_2_ DRX cathodes through the introduction of cation vacancies into the lattice, demonstrating Zn^2+^ (de)intercalation with a reversible capacity of approximately 170 mAh g^−1^.

We start this work with a systematic investigation of the possibility of bulk Zn‐ion diffusion in a series of novel Zn‐based DRX materials with the general formula Zn_x_TM_2‐x_O_2_ (TM = Mn, Fe, Co, Ni, Mg, and Cu) in nonaqueous ZIBs. All of these materials exhibited negligible electrochemical activity, as consistently confirmed by electrochemical performance (< 10 mAh g^−1^), ex situ x‐ray diffraction (XRD), and x‐ray absorption near‐edge structure (XANES) spectroscopy.

Turning to modeling for insights, our density functional theory (DFT) calculations reveal that Zn^2+^ faces prohibitively high migration barriers at the atomic scale, even through the 0‐TM channels. This indicates that Zn^2+^ migration is intrinsically sluggish at the microscopic level and therefore incapable of forming a macroscopic percolation network required for long‐range transport. However, our DFT modeling shows that the presence of cation vacancies at octahedral sites significantly reduces the Zn^2+^ migration barrier during hopping through interstitial tetrahedral sites. To realize such a vacancy enriched system experimentally, we turn to fully delithiated Mn‐based DRX compounds which inherently contain cation vacancies generated by Li deintercalation. We use these cation vacancy‐rich MnO_2_ and Mn_0.4_Ti_0.4_O_2_ DRX materials to successfully demonstrate reversible high‐capacity cycling in a nonaqueous Zn battery cell, and thus demonstrate a vacancy‐engineering strategy for enabling multivalent cation diffusion in oxide cathode materials.

## 2 Results and Discussion

**FIGURE 1:**
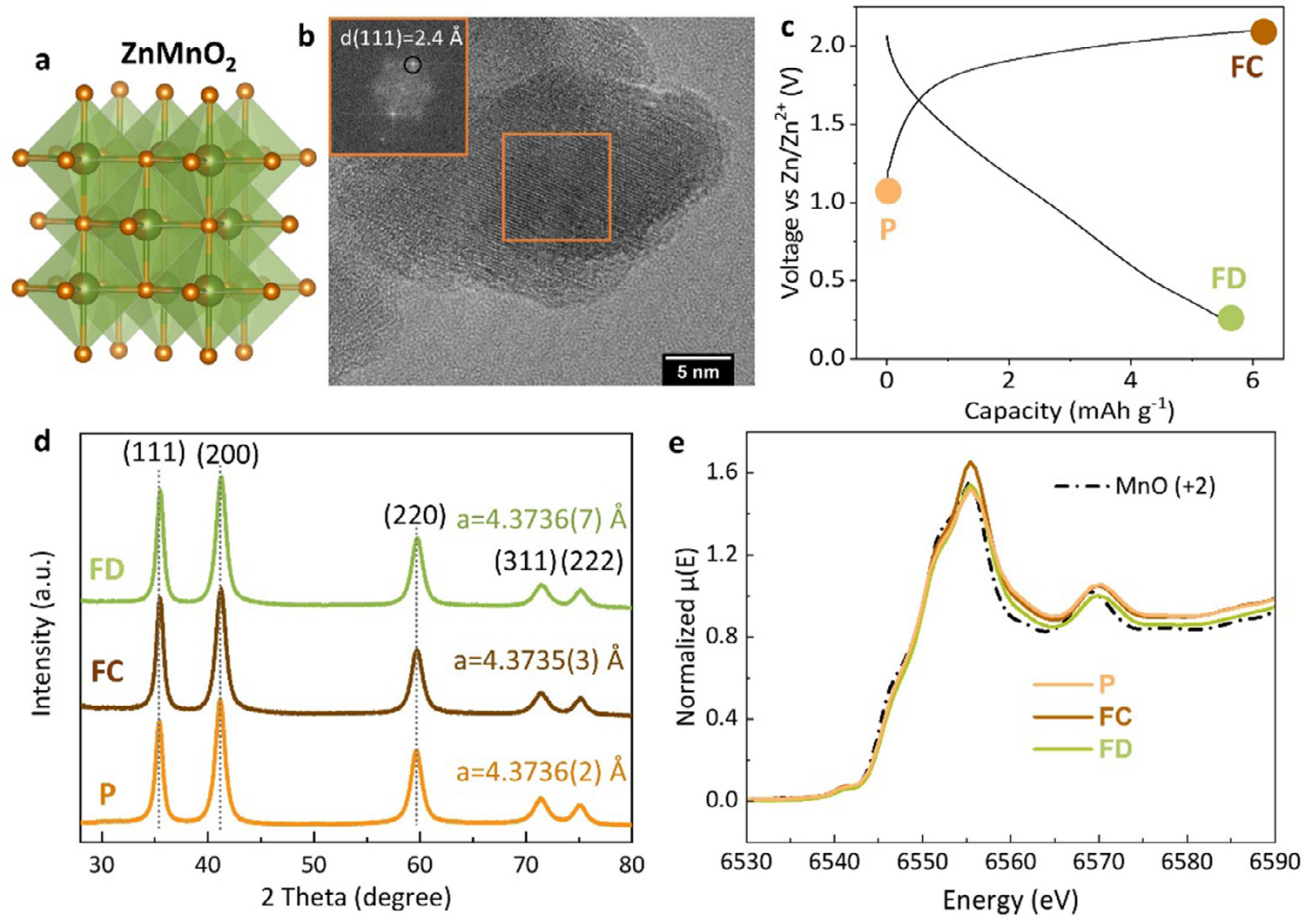
(a) Schematic of DRX ZnMnO_2_. Orange atoms represent oxygen, while brown and green atoms illustrate the random distribution of Zn and Mn cations. (b) HR‐TEM image of a pristine ZnMnO_2_ particle. (c) Galvanostatic load curve of ZnMnO_2_ in nonaqueous electrolyte of 1 M Zn(TFSI)_2_ in acetonitrile at 10 mA g^−1^. (d) Ex situ XRD patterns of ZnMnO_2_ powder at different states of charge: P (pristine), FC (fully charged), and FD (fully discharged), with corresponding points indicated in (c). Unit cell parameters were obtained from Rietveld refinement. (e) Ex situ Mn K‐edge XANES spectra of ZnMnO_2_ at P, FC, and FD states, with MnO as a reference.

A family of Zn‐based disordered rocksalt (DRX) materials was explored to assess their viability as cathodes in nonaqueous ZIBs. As a starting point, we first investigated the previously reported ZnMnO_2_ (ZMO), which was synthesized via high‐energy ball milling. The resulting material crystallizes in a cubic DRX structure, where oxygen atoms form a face‐centered cubic lattice, and Zn and Mn are randomly distributed over the octahedral sites (Figure 1a). Owing to the high‐energy ball milling process, the particles exhibit sizes in the range of 50–300 nm, as observed by scanning electron microscope (SEM) (Figure S1), while high‐resolution transmission electron microscopy (HR‐TEM) reveals crystalline domains of only 5–10 nm (Figure 1b).

However, electrochemical testing of DRX ZMO with a 1 M Zn(TFSI)_2_ in acetonitrile electrolyte showed negligible capacity (Figure 1c), indicating that the material is electrochemically inactive toward Zn extraction or insertion. To further substantiate this conclusion, ex situ X‐ray diffraction (XRD) (Figure 1d) was performed on ZMO in the pristine (P), fully charged (FC), and fully discharged (FD) states. The diffraction patterns exhibited no detectable shift in peak positions or variation in lattice parameters, implying the absence of lattice contraction or expansion typically associated with Zn deintercalation or intercalation. Complementing Mn XANES analysis (Figure 1e) revealed no detectable shift in the absorption edge across the different electrochemical states, with spectra remaining nearly identical to that of reference MnO (Mn^2+^). Collectively, these results confirm that DRX ZMO is electrochemically inactive in nonaqueous electrolyte and does not accommodate Zn‐ion transport within its lattice.

The poor electrochemical performance of the ZMO cathode in nonaqueous electrolytes contrasts with its behavior in aqueous systems, where it delivers a high charge capacity of 312 mAh g^−1^, as reported in our previous study [16]. However, in both electrolyte environments, the capacity associated with Zn^2+^ (de)intercalation in the DRX lattice remains limited, indicating that the lack of Zn mobility is largely independent of the electrolyte and is instead a consequence of the cathode structure. In aqueous ZIBs, the presence of H^+^ enables DRX ZMO to undergo self‐dissolution, and the dissolved Mn species can be redeposited onto the cathode during charging, resulting in charge storage dominated by a Mn dissolution–redeposition mechanism. This process is largely insensitive to the cathode structure. In contrast, in nonaqueous electrolytes, charge storage relies exclusively on solid‐state Zn^2+^ migration within the cathode lattice, which is dependent on a suitable host structure to facilitate ion migration. This fundamental difference explains why a wide range of Mn‐based materials exhibit high capacities in aqueous ZIBs but much lower capacities in nonaqueous ZIBs. For example, α‐MnO_2_ delivers ∼270 mAh g^−1^ in aqueous electrolytes but only ∼45 mAh g^−1^ in nonaqueous electrolytes [17], underscoring the intrinsic challenge of developing intercalation‐type cathodes compatible with nonaqueous multivalent batteries.

In general, DRX oxides are known to possess intrinsically poor electronic conductivity, which can significantly hinder coupled ion–electron transport during (de)intercalation [18, 19]. To enhance the electronic conductivity of the cathode mixture, ZnMnO_2_ powder was co‐milled with conductive carbon to ensure intimate particle–carbon contact to form a uniform conductive coating. Despite this, electrochemical testing again revealed negligible reversible capacity (Figure S2a), while Mn K‐edge XANES (Figure S2b) confirmed the invariance of the Mn valence state during cycling. These results indicate that the absence of electrochemical activity arises from factors other than electronic limitations.

To determine whether the observed electrochemical inactivity of ZnMnO_2_ is specific to Mn or a more general feature of Zn‐based DRX oxides, we extended our investigation to other TM systems. Insights from analogous stoichiometric Li‐based DRX compounds (LiTMO_2_, TM = Ni, Mn, Co, Fe, and Ti) have shown that electrochemical performance varies strongly with the TM species [20], likely due to differences in compound electronic structure, local bonding environments and the extent of short‐range ordering (SRO) [21]. Following this rationale, stoichiometric ZnTMO_2_ compounds (TM = Fe, Co, and Ni) were synthesized and evaluated in the same nonaqueous electrolytes. All of them exhibited negligible electrochemical capacity (Figure S3), demonstrating that the lack of electrochemical activity is not unique to Mn. Moreover, motivated by reports that high‐entropy strategies can suppress SRO and enhance Li^+^ transport [22], a high‐entropy Zn‐based DRX oxide, ZnMg_0.25_Cu_0.25_Co_0.25_Ni_0.25_O_2_ was synthesized and tested. However, this material likewise showed negligible electrochemical activity (Figure S4).

Another important factor to consider is the formation of macroscopic percolation pathways. In Li‐rich DRX compounds, long‐range Li^+^ transport is enabled by interconnected 0‐TM channels. A 0‐TM channel refers to a migration pathway for Li^+^ ions that involves no face‐sharing TM ions for the interstitial tetrahedral sites. Increasing the Li:TM ratio introduces more 0‐TM sites, thereby enhancing the probability of forming percolating networks and thus facilitating long‐range Li^+^ diffusion [23, 24].

Building on this principle, Zn‐rich DRX oxides were synthesized and evaluated. Attempts to synthesize Mn‐ and Fe‐based DRX oxides were stymied by the large size mismatch between Zn^2+^ and Mn^2+^/Fe^2+^ [25], preventing the formation of pure Zn‐rich DRX phases, and instead resulting in prevalent ZnO impurities in the final product. However, the use of the smaller ionic radii transition metals of Co^2+^ and Ni^2+^ did allow for the successful preparation of Zn‐rich DRX structures. Stable compositions such as Zn_1.2_Co_0.8_O_2_, Zn_1.2_Ni_0.8_O_2_, and Zn_1.4_Ni_0.6_O_2_ were successfully synthesized (Figure S5). These Zn‐rich phases, in principle, should provide a higher concentration of 0‐TM sites, thereby increasing the probability of forming percolating pathways for Zn^2+^. However, electrochemical testing revealed insignificant capacity in all cases.

Collectively, these results all demonstrate that Zn‐based DRXs remain electrochemically inactive regardless of the DRX composition (including TM species, configurational entropy, and Zn excess content), the electrolyte type, and the electronic conductivity of the cathode composite. This strongly suggests that the fundamental limitation arises from restricted Zn^2+^ diffusion at the microscopic atomic scale rather than insufficient macroscopic percolation.

**FIGURE 2:**
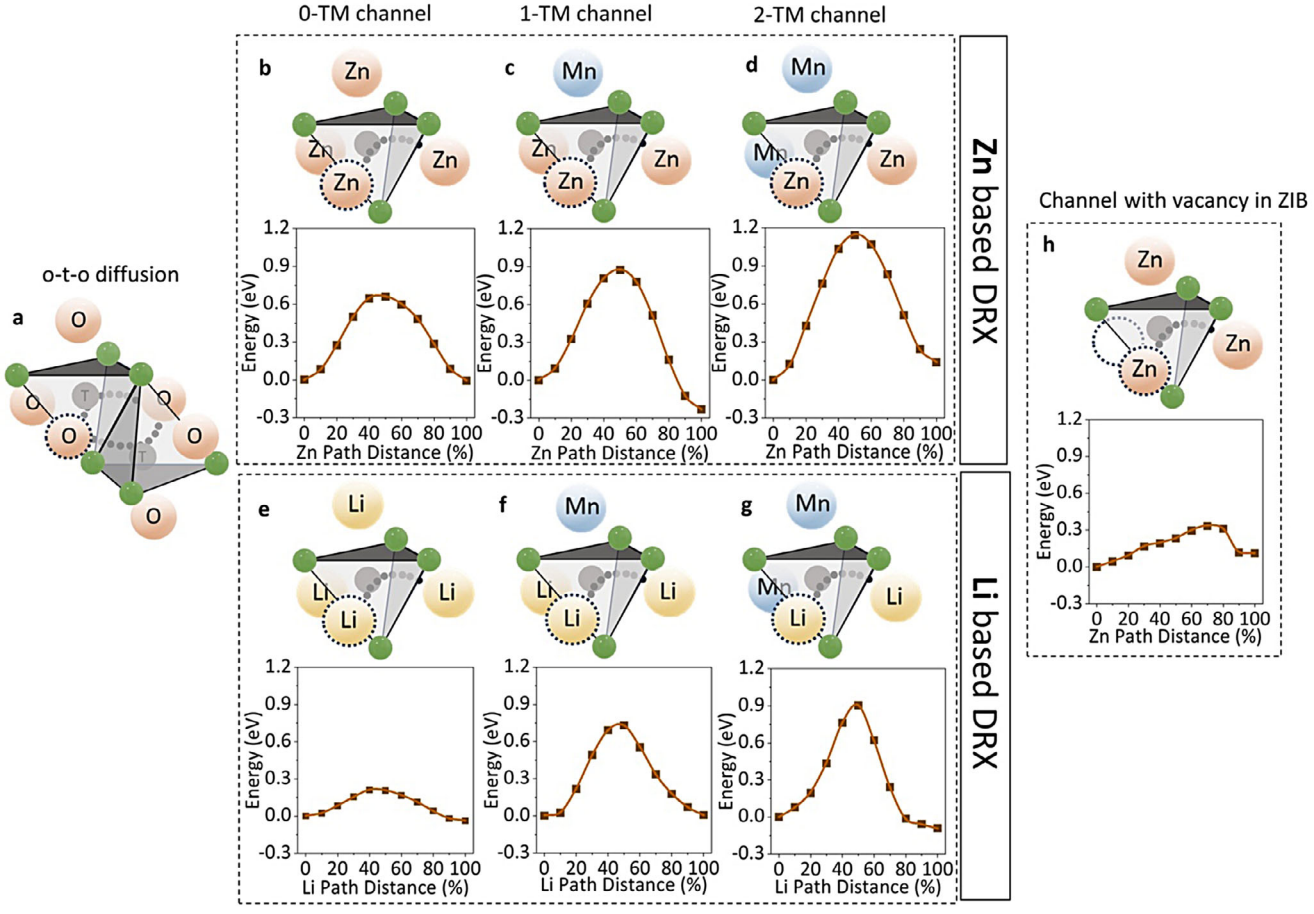
(a) Schematic of ion diffusion through an octahedral–tetrahedral–octahedral (o–t–o) channel in a DRX unit cell. Green atoms represent oxygen. An O represents octahedral sites while a T represents a tetrahedral site. (b)–(d) Calculated migration barriers for 0‐TM, 1‐TM, and 2‐TM channels with Mn^2+^ as the transition metal and Zn^2+^ as the charge carrier in Zn‐based DRX. (e)–(g) Calculated migration barriers for 0‐TM, 1‐TM, and 2‐TM channels with Mn^3+^ as the transition metal and Li^+^ as the charge carrier in Li‐based DRX. (h) Migration barrier of Zn^2+^ in the 0‐TM channel with one face sharing vacancy.

Previous studies have shown that charge carriers hopping between two octahedral sites in a rocksalt structure pass through an intermediate tetrahedral site, forming an octahedral–tetrahedral–octahedral (o–t–o) pathway as shown in Figure 2a. The activation energy of charge carrier hopping is largely associated with the tetrahedral intermediate, which is primarily determined by electrostatic repulsion between the migrating ion in the tetrahedral site and the species occupying the two face‐sharing octahedral sites [23].

To gain insight into Zn^2+^ transport in DRX oxides, Zn^2+^ migration barriers were calculated using DFT for three representative pathways: 0‐TM, 1‐TM, and 2‐TM channels. Due to the disordered arrangement of cations in DRX, numerous possible ion migration configurations exist. In this work, we focus on one specific case each. When Zn migrates between two certain adjacent octahedral sites, it may pass through one of the two intermediate tetrahedral sites, as shown in Figure S6. We assume one tetrahedral site is surrounded by two TM atoms, making it a less favorable migration path. The other tetrahedral sites, with 0‐TM, 1‐TM, and 2‐TM pathways, are less hindered, making Zn migration more favorable through these interstitial sites.

The calculated migration barriers for the representative pathways shown in Figure 2b–d reveal severe transport limitations for Zn^2+^. Even for the most favorable 0‐TM channel case, the barrier remains high at 0.67 eV (Figure 2b), increasing further to 1.11 eV for the 1‐TM channel and to 1.14 eV for the 2‐TM channel (Figure 2d). Since both Zn^2+^ and Mn^2+^ are divalent, the increase from the 0‐TM pathway to the 2‐TM pathway cannot be attributed to electrostatic repulsion force alone. Unlike the largely ionic Zn─O interactions, Mn─O bonds involve stronger covalency, which locally stiffens the oxygen framework and reduces its ability to undergo the cooperative distortions required for Zn transport [26]. Even in the nominally hopping favorable 0‐TM channel, the migration barrier remains too high to support appreciable Zn mobility. This inherent limitation clarifies why even in Zn‐rich DRX compositions, despite statistically providing more 0‐TM pathways, measurable electrochemical activity is still not observed.

We used the same calculation methodology to evaluate the case for Li^+^ migration barriers, which are known to be able to diffuse through these materials. The calculations support this expectation (Figure 2e–g). The 0‐TM channel for Li^+^ exhibits a much lower barrier of 0.25 eV (Figure 2e), which is consistent with previously reported values for 0‐TM channels in Li‐rich DRX (∼0.29 eV for Li^+^ hopping) [15]. For the 1‐TM channels, the Li migration barriers increase significantly to above 0.73 eV. In the 2‐TM channels, the barriers rise further to 1.00 eV. Notably, the relaxed unit‐cell parameter of Li‐based DRX (4.22 Å, close to the experimental value of 4.17 Å in Figure S9) is smaller than that of Zn‐based DRX (4.45 Å, close to the experimental value of 4.37 Å in Figure 1d). Thus, the tetrahedral size for Li^+^ migration is smaller than that for Zn^2+^, yet the Li^+^ migration barrier in 0‐TM channel is significantly lower than that of Zn^2+^. This suggests that the size of the tetrahedral interstitial sites in Zn‐based DRX does not impede ion diffusion channels. Instead, the limitations arise from the divalent nature of Zn^2+^ and the electrostatics, which fundamentally restricts its local hopping. Because Zn^2+^ transportation is constrained at an atomic level, even with tailored local order, Zn^2+^ cannot form a continuous macroscopic percolation network for long‐range migration, rendering DRX structures electrochemically inactive for Zn storage.

Cation disorder creates diverse local environments, which significantly alter the site energy for charge carriers. Consequently, the migration barrier is sensitive to these local fluctuations, leading to variations in energy between initial and final positions. While our current DFT calculations provide fundamental insights into Zn^2+^ and Li^+^ transport, they represent only a specific local configuration. A comprehensive analysis of migration barrier distributions would require extensive sampling across the wide range of local environments created by cation disorder [27].

However, even within these specific local configurations, a clear trend emerges: the electrostatic interactions between divalent Zn^2+^ and surrounding cations represent a primary bottleneck for migration. Our results suggest that minimizing these interactions, specifically during the transition from octahedral to tetrahedral sites, is a critical factor for enabling transport. We propose introducing cation vacancies into the DRX lattice to achieve this. DFT calculations revealed that, in the 0‐TM channel, replacing one face‐sharing Zn^2+^ ion with a vacancy can lower the barrier from 0.67 eV to 0.33 eV (Figure 2h), significantly promoting the favorability of local Zn^2+^ migration. Physically, the introduction of cation vacancies can reduce the migration barrier through two cooperative effects. First, removing a neighboring cation decreases the strong electrostatic repulsion experienced by the migrating Zn^2+^ ion at the high‐energy tetrahedral transition state, thereby stabilizing the intermediate configuration. Second, the presence of vacancies provides additional local free volume and structural flexibility, allowing the surrounding oxygen framework to relax more readily during Zn^2+^ migration. Together, these effects substantially lower the transition‐state energy for Zn^2+^ hopping. Accordingly, a sufficiently high concentration of cation vacancies in the DRX lattice can potentially enable bulk Zn^2+^ (de)intercalation through the formation of a percolating network of low‐energy migration pathways.

Attempts to synthesize vacancy‐containing Zn‐based DRX materials, such as Zn_0.5_MnO_2_ and Zn_0.75_FeO_2_, were first explored. Introducing Zn vacancies necessitates adjusting the transition‐metal valence states by reducing the proportion of rocksalt precursors (e.g., MnO and FeO) and incorporating higher‐valence sources such as MnO_2_ and Fe_2_O_3_. Unfortunately, these compositional adjustments destabilize the DRX framework, resulting in impurity phase formation (Figure S7) due to an insufficient amount of rocksalt component to sustain the rocksalt structure.

**FIGURE 3:**
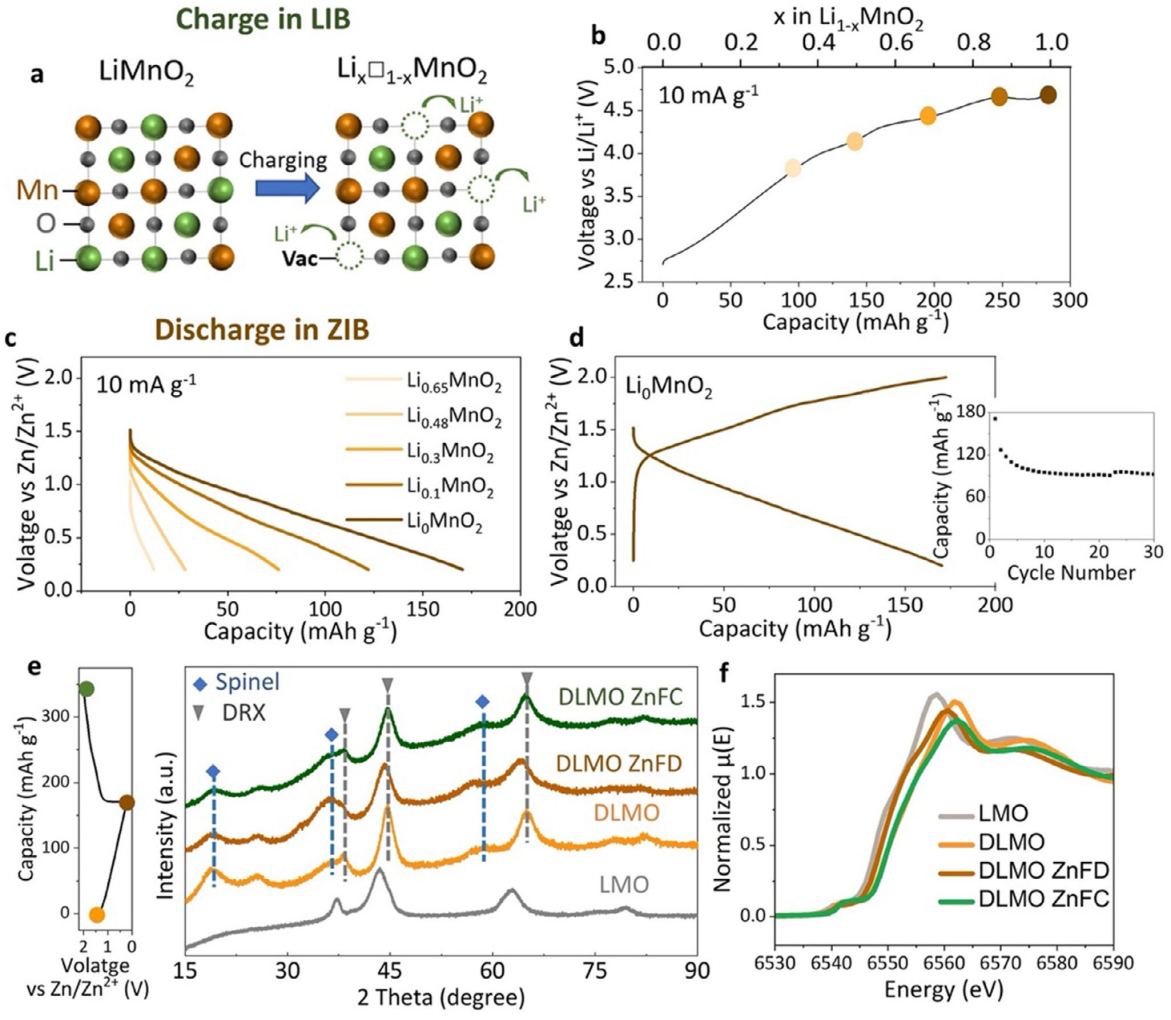
(a) Schematic of delithiation of DRX LiMnO_2_ (LMO) in a Li‐ion battery to generate a vacant DRX structure. (b) Charge curve of LMO in a Li‐ion battery at 10 mA g^−1^. (c) Discharge capacities of partially delithiated LMO, tested in nonaqueous ZIBs at 10 mA g^−1^; the corresponding delithiation states are labelled in panel (b). (d) Charge–discharge profile of delithiated LMO (DLMO) in a Zn‐ion battery, with inset showing cycling performance at 10 mA g^−1^. (e) Ex situ powder XRD and (f) ex situ Mn K‐edge XANES spectra of LMO, DLMO, DLMO ZnFD, and DLMO ZnFC. DLMO ZnFD represents the first full discharge state in ZIBs, and DLMO ZnFC represents the first full charge state in ZIBs.

A different strategy was employed to introduce vacancies into the DRX framework. Instead of directly synthesizing Zn‐based DRX with intrinsic vacancies, a delithiated Li‐based DRX was used as a model system. DRX LiMnO_2_ (LMO) was selected for its structural and compositional similarity to ZnMnO_2_. The LMO was electrochemically charged in a Li cell to various extents, generating controlled concentrations of cation vacancies (Figure 3a,b). The resulting delithiated electrodes were then discharged in Zn‐ion cells to assess their Zn‐hosting capability. As shown in Figure 3c, higher vacancy concentrations in the cathode significantly enhanced Zn insertion, confirming that vacancies are critical for facilitating Zn storage. As shown in Figure 3d, fully delithiated DRX LiMnO_2_ (DLMO) discharged in a Zn‐ion cell delivered a discharge capacity of ∼170 mAh g^−1^. The following charge process recovered nearly the same capacity, confirming the reversibility of Zn insertion and extraction. With extended cycling, the capacity gradually declined, yet after 30 cycles the material still retained ∼85 mAh g^−1^.

To understand the structural evolution, ex situ XRD measurements were performed on LMO at different charge states. As shown in Figure 3e, in DLMO, after delithiation, the DRX peaks shift to higher angles, consistent with lattice contraction associated with Li extraction. Notably, additional peaks appear, which can be indexed to a spinel phase [28]. Rietveld refinement shows that the spinel fraction increases progressively with charging, reaching ∼40 wt% at the top of charge in the DLMO samples (Figure S8). The phase transformation observations align with the widely reported phase evolution in Mn‐based oxide cathodes [29, 30, 31], including monoclinic and orthorhombic LiMnO_2_, [30] where the highly mobile Mn^3+^ ions facilitate Mn migration [28], resulting in the formation of the thermodynamically favored spinel phase. This coexistence of spinel and DRX structures enables the near‐complete extraction of Li in the DRX LMO compound.

When the DLMO was subsequently tested in a Zn‐ion cell, ex situ XRD results (Figure 3e) showed that both the spinel peak at ∼58° and the DRX peak at ∼65° shifted to lower angles during Zn insertion. This systematic peak shift corresponds to lattice expansion, indicating that Zn ions were incorporated into both the spinel and DRX phases. After charging, these peaks returned to their original positions, confirming the reversibility of Zn intercalation and deintercalation in both phases. Moreover, the spinel phase persisted throughout the entire discharge/charge process in the Zn‐ion cell, as confirmed by the refinements of diffraction data at different states of charge (Figure S9). Complementary Mn K‐edge XANES analysis (Figure 3f) revealed that the Mn was reduced during Zn insertion. However, the valence change did not fully return to that of pristine LMO, consistent with the lower Zn discharge capacity (∼170 mAh g^−1^) compared to the Li charging capacity (∼285 mAh g^−1^). Upon re‐charging in the Zn cell, the Mn valence state recovered, demonstrating the reversibility of the redox process, and confirming reversible Zn (de)intercalation.

The challenge, however, with this in situ‐formed spinel phase in DLMO is that it makes it difficult to isolate and understand Zn transport behavior within the DRX structure alone. Nevertheless, these results demonstrate that once vacancies are introduced, Zn diffusion in DRX can be unlocked and both DRX and spinel phases can participate in reversible Zn (de)intercalation. To examine whether our proposed vacancy strategy is effective for other compositions, we extended our study to other Mn‐based DRX systems, including Li‐rich Li_4_Mn_2_O_5_ (LMO425) and Na‐based NaMnO_2_ (NMO), both synthesized by ball milling with particle sizes of ∼50–300 nm, comparable to LMO (Figure S10). Similar to LMO, both LMO425 (Figures S11 andS12) and NMO (Figures S13 and S14) also undergo structural transformation to spinel upon delithiation/desodiation. When these delithiated and desodiated materials were cycled in Zn‐ion cells, both the DRX and the in situ‐formed spinel phases became electrochemically active (Figures S11 and S13). During Zn intercalation and deintercalation, both phases exhibited reversible structural changes, as confirmed by XRD (Figures S11c and S13c), along with reversible Mn valence changes, as evidenced by XANES (Figures S11d and S13d).

**FIGURE 4:**
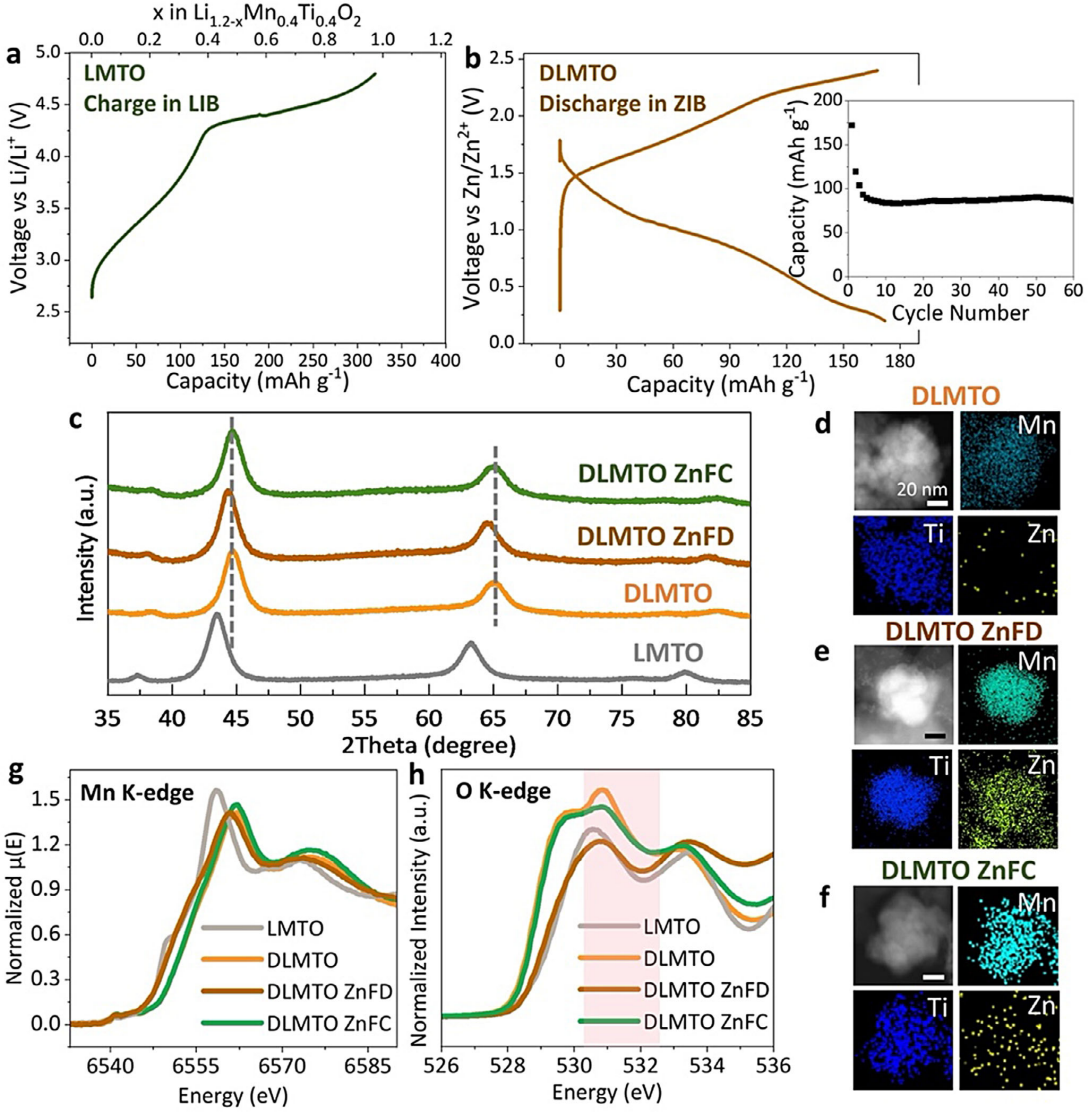
(a) Charge curve of Li_1.2_Mn_0.4_Ti_0.4_O_2_ (LMTO) DRX (ball‐milled at 600 rpm for 6 h) in a Li‐ion battery at 10 mA g^−1^. (b) Discharge–charge profile of delithiated LMTO (DLMTO) in a Zn‐ion battery, with inset showing cycling stability at 10 mA g^−1^. (c) Ex situ powder XRD of LMTO, DLMTO, DLMTO ZnFD, and DLMTO ZnFC. (d)–(f) STEM–EDS mapping of Mn, Ti, and Zn in DLMTO, DLMTO ZnFD, and DLMTO ZnFC. (g) Ex situ Mn K‐edge XANES spectra and (h) O K‐edge XAS spectra collected in bulk‐sensitive TFY mode of LMTO, DLMTO, DLMTO ZnFD, and DLMTO ZnFC. DLMTO ZnFD represents the first full discharge state in ZIBs, and DLMTO ZnFC represents the first full charge state in ZIBs.

To understand Zn transport behavior within the DRX structure alone, a more stable DRX framework that suppresses spinel phase transformation in the early cycle is required. In Li‐based DRX systems, it is well established that incorporating at least one d^0^ transition metal is beneficial to stabilize the structure [32]. d^0^ cations preferentially occupy highly distorted octahedral sites, leaving the less distorted sites available for cations with partially filled d orbitals, thereby lowering the overall energy of the disordered structure [33, 34]. The d^0^ cation, Ti^4+^ was introduced to synthesize Li_1.2_Mn_0.4_Ti_0.4_O_2_ (LMTO) as a model system. Unlike the other DRX materials discussed in this work, which were prepared by high‐energy ball milling and naturally adopt particle sizes of ∼50–300 nm, this material was synthesized via a solid‐state route and exhibited large particles (∼5–10 µm), as shown in Figure S15a. To reduce the Li^+^ diffusion length in LMTO, the material was subsequently processed by ball milling at 600 rpm for 6 h, reducing the particle size to ∼50–300 nm (Figure S15c). In Li‐ion cells, this nanosized material delivered a charge capacity of ∼320 mAh g^−1^ (Figure 4a), in good agreement with previous reports [35].

When the delithiated Li_1.2_Mn_0.4_Ti_0.4_O_2_ (DLMTO) cathode was assembled in ZIBs, it delivered a reversible high capacity of approximately 170 mAh g^−1^, with cycling behavior as shown in the inset of Figure 4b. To benchmark the electrochemical performance of the DRX cathode, DLMTO was compared with several representative cathode materials previously reported for the nonaqueous ZIB system, as summarized in Table S1. DLMTO delivers a relatively high reversible capacity compared with several representative cathodes such as δ‐MnO_2_ [10], γ′‐V_2_O_5_[9], and VS_2_ [11]. In addition, its operating voltage range is comparable to that of δ‐MnO_2_ [10], although lower than that of spinel ZnNi_1/2_Mn_1/2_CoO_4_ [36] and K_1.6_Mn_1.2_Fe(CN)_6_ [37], PBA materials. The cycling stability of DLMTO remains limited, indicating that further optimization is still needed.

Several factors may contribute to the observed capacity decay in both DLMTO and the aforementioned DLMO systems. First, the relatively high operating voltage may accelerate electrolyte decomposition and destabilize the cathode–electrolyte interface. These parasitic interfacial reactions are likely further exacerbated by the nanosized DRX particle morphology [38]. Second, Mn^3+^ formed during cycling in these Mn‐based DRX would undergo Jahn–Teller distortion and disproportionation reactions, promoting Mn dissolution and subsequent capacity fading [39]. Third, the short‐range ordering may gradually evolve during repeated Zn^2+^ insertion/extraction, potentially disrupting the percolating Zn^2+^ diffusion network. Similar behavior has also been reported in DRX materials for Li‐ion batteries [40]. Future optimization may include surface protection, electrolyte engineering, and compositional tuning to suppress interfacial side reactions, mitigate Mn dissolution, and stabilize the vacancy‐assisted Zn^2+^ diffusion network during cycling.

Figure 4c shows that the inclusion of Ti^4+^ ensures that the DLMTO can maintain a single‐phase DRX structure without the formation of secondary phases, as intended. Subsequently, all the DRX peaks shift reversibly during Zn insertion and extraction, corresponding to unit cell expansion and contraction, as confirmed by Rietveld refinement in Figure S16.

According to the capacity measurements, at full discharge, the composition reached Zn_0.25_Li_0.23_□_0.72_Mn_0.4_Ti_0.4_O_2_, indicating that a substantial fraction of cation vacancies still remained. Less than half of the vacancies in DLMTO are filled, most likely because the residual vacant sites are required to sustain Zn^2+^ transport. Once too many of these vacancies are occupied, electrostatic repulsion of local Zn hopping would rise sharply and the long‐range migration pathways would be locked, making Zn storage kinetically inaccessible, as suggested by the DFT modeling results in Figure 2. This is similar to pristine ZnMnO_2_, where a fully cation‐occupied DRX framework is incapable of reversible Zn^2+^ (de)intercalation.

It is also interesting to note that the lattice parameter of DLMTO (4.05 Å) is substantially smaller than that of ZMO (4.37 Å), yet DLMTO exhibits much more pronounced Zn storage and transport capability compared to ZMO. This contrast further underscores that the key to enabling Zn mobility in the DRX framework is the introduction of vacancies instead of intermediate tetrahedral sites.

To elucidate the charge compensation mechanism involved, ex situ Mn K‐edge XANES and O–K edge soft x‐ray absorption (XAS) spectroscopy were collected at different states of charge, as LMTO is an archetypal Li‐rich DRX that exhibits both Mn‐ and O‐redox. As shown in Figure 4g, after delithiation, the Mn K‐edge position of DLMO shifts to higher energy relative to pristine LMTO, suggesting Mn^3+^/Mn^4+^ oxidation. When DLTMO is cycled in a nonaqueous ZIB, reversible Mn reduction and oxidation are consistently observed. To probe the oxygen contribution, soft O–K edge XAS in total fluorescence yield (TFY) mode was performed. Figure 4h displays the O–K pre‐edge feature, which originates from O 1s to 2p transitions and reflects the density of Mn 3d‐O 2p hybridized states. The emergent feature centered around 529 eV in charged DLMTO can be attributed to characteristics of Mn^4+^ oxides [35], supporting the presence of Mn^3+^/Mn^4+^ oxidation during charging. Another increase in the pre‐edge intensity at around 530.5–532.5 eV (pink shaded region in Figure 4h) is observed, indicative of electron hole formation on oxygen, and in line with previously reported O‐redox behavior in Li‐rich DRX materials [35, 41].

Given the reversible shifts in both Mn and O K‐edge spectra and the close spectra similarity between DLMTO and first cycle full charged DLMTO (DLMTO ZnFC), we conclude that both Mn‐ and O‐redox processes contribute to the capacity during Zn^2+^ (de)intercalation into the DRX material. This finding provides evidence that O‐redox chemistry is accessible beyond Li‐ and Na‐ion batteries and suggests its potential for developing high‐voltage cathodes in ZIBs. To study any potential O_2_ evolution arising from O‐redox, differential electrochemical mass spectrometry (DEMS) was employed to monitor gas evolution in real time during cycling. As shown in Figure S17, negligible oxygen evolution was observed during the initial discharge and charge process in ZIBs. This is likely because irreversible O_2_ release in oxygen‐redox cathodes primarily occurs during the first charge process, corresponding to the initial delithiation step in Li‐ion batteries, where lattice oxygen redox can result in irreversible gaseous O_2_ loss. In subsequent cycles, oxygen redox can instead proceed through the formation of bulk‐confined O^n−^ species, which generally do not lead to significant gaseous O_2_ evolution [42, 43].

Lastly, scanning transmission electron microscopy–energy‐dispersive x‐ray spectroscopy (STEM–EDS) mapping was performed on DLMTO, DLMTO‐ZnFC, and DLMTO‐ZnFD (Figure 4d–f). Peaks at signature emission energies for Zn were observed at the nanoscale during discharge, which then disappeared following charging, reinforcing the conclusion that Zn insertion and extraction are highly reversible at the bulk particle level. Taken together, these experimental observations are in strong agreement with the DFT modeling results, demonstrating that introducing cation vacancies can largely activate the DRX framework to enable facile Zn^2+^ intercalation.

To assess the particle‐size dependence of ion transport, we first examined Li^+^ diffusion in the DRX framework. LMTO was ball milled under milder conditions (300 rpm, 6 h), producing submicron particles (∼200–1000 nm) as shown in Figure S15b. When tested in Li‐ion cells, this material delivered a charge capacity of ∼315 mAh g^−1^ (Figure S18a), comparable to the ∼320 mAh g^−1^ achieved with the finer nanosized material prepared at 600 rpm for 6 h (Figure 4a). This indicates that Li^+^ diffusion can still occur effectively even within these larger submicron particles. In contrast, when cycled in Zn‐ion cells, the same submicron material LMTO (ball milled 300 rpm, 6 h) delivered a reversible capacity of only ∼80 mAh g^−1^ (Figure S18b), far below the 170 mAh g^−1^ obtained with the finer particles produced under harsher milling conditions (600 rpm, 6 h) (Figure 4b). These results further highlight that beyond introducing cation vacancies, reducing diffusion lengths within DRX crystallites and increasing interfacial contact with the electrolyte are also necessary to achieve effective electrochemical utilization.

To directly compare the diffusion behavior of Li^+^ and Zn^2+^ in DRX materials, galvanostatic intermittent titration technique (GITT) measurements were carried out on LMTO prepared under harsher ball‐milling conditions (600 rpm, 6 h). It is important to note that, unlike the local ion hopping barrier obtained from DFT modeling, GITT captures macroscopic diffusion at the electrode level, integrating contributions from bulk material diffusion, grain boundaries, particle interfaces, and percolation pathways in the cathode composite. To focus on bulk ion diffusion, the same host material, DLMTO, was used as a starting point for subsequent ion‐insertion experiments with either Li^+^ or Zn^2+^. As discussed before, DLMTO exhibits similar phase evolution and redox processes upon accommodating Zn^2+^ and Li^+^. To allow for robust comparison in the GITT measurements, the same cutoff capacity (170 mAh g^−1^) was applied to both systems, thereby minimizing potential interference arising from probing different redox processes.

**FIGURE 5:**
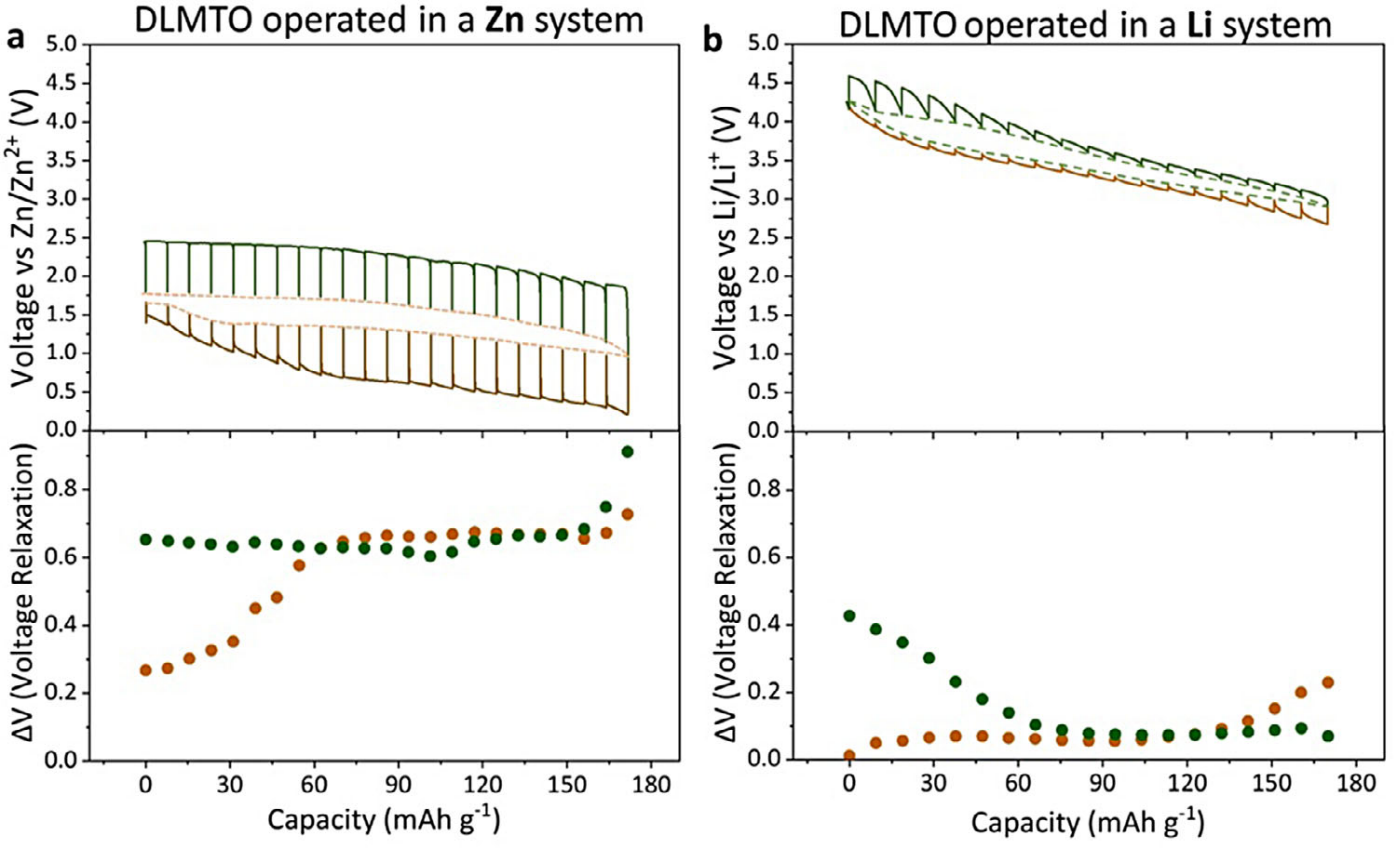
Galvanostatic intermittent titration technique (GITT) profiles of DRX DLMTO (ball‐milled at 600 rpm for 6 h) measured at 10 mA g^−1^ with 1 h current pulses followed by 4 h relaxation to reach equilibrium, tested in (a) a Zn‐ion battery and (b) a Li‐ion battery. The change of voltage after relaxation (Δ*V*) is plotted against the capacity before relaxation. The orange data represent the discharge process, while the green data represent the charge process.

Under these controlled conditions, the GITT measurements revealed a clear kinetic contrast between Li^+^ and Zn^2+^ (de)intercalation. As shown in Figure 5b, Li^+^ insertion/extraction shows a small voltage hysteresis of only ∼0.3 V. In contrast, Zn^2+^ insertion/extraction exhibited a much larger voltage hysteresis of ∼1.6 V (Figure 5a), despite being tested at the same low current density (10 mA g^−1^). By tracking the quasi‐equilibrium potential after each voltage relaxation (dashed lines in Figure 5a), it was observed that in the Zn‐ion cell, the quasi‐equilibrium discharge and charge curves closed most of the hysteresis gap. This indicates that the large voltage hysteresis is primarily due to slow Zn^2+^ insertion and extraction kinetics, which can reduce the round‐trip energy efficiency of the battery system. The pronounced polarization observed with Zn^2+^ cycling indicates the need for higher voltages to fully extract Zn^2+^ during charging. This high voltage brings challenges for the electrolyte stability and can exacerbate degradation of cathode/electrolyte interface during cycling. Whereas for Li^+^ (Figure 5b), the quasi‐equilibrium potential only slightly narrowed the gap between the charge and discharge curves, indicating much faster Li^+^ ion transport and lower kinetic barriers.

The differences between the diffusion kinetics of the two cations was further highlighted by the magnitude of the voltage relaxation (∆*V*) extracted from the GITT curves, as displayed in the lower panel of Figure 5. DLMTO operated in a ZIB exhibits a large voltage relaxation of up to 0.95 V, whereas in a LIB the overpotential remains significantly lower throughout the entire cycle. To further validate this kinetics analysis, GITT measurements with shorter pulse durations (10 min) were performed, as shown in Figure S19. The extracted voltage relaxation values confirm that the polarization associated with Zn^2+^ diffusion in DLMTO is significantly larger than that for Li^+^ in DLMTO, consistent with the data in Figure 5. It should be noted that diffusion coefficients were not calculated from the GITT data. Due to the nanoscale particle size of the DRX material (∼50–300 nm), the pulse duration required to satisfy the semi‐infinite diffusion assumption (*τ* ≪ L^2^/D) to calculate the diffusion coefficients is expected to be only a few seconds (∼5–20 s) or even shorter [44]. At such short timescales, the voltage response is dominated by the instantaneous IR drop and instrumental noise rather than diffusion‐controlled polarization. Consequently, extracting reliable diffusion coefficients from GITT could result in overinterpretation of the data. Therefore, only the voltage relaxation is discussed here as a qualitative indicator of ion‐transport kinetics.

Up to this point, we have examined how the number and type of neighboring cations (i.e., cation vacancy concentration) influences Zn migration. The coulombic repulsion experienced by Zn^2+^ diffusion through the o–t–o pathway is also influenced by the distance between the migrating Zn and its surrounding cations, as well as the chemical identity of the anions. In this context, sulfide‐based DRX materials, specifically LiTiS_2_ (LTS), were investigated. The larger S^2−^ anions expand the whole DRX lattice and the associated tetrahedral interstices (Figure S20a), providing additional space for ion relaxation at the transition state, which is expected to reduce electrostatic repulsion and lower Zn migration barrier, thereby enabling more facile Zn^2+^ transport. In addition, the softer (less electronegative) and more polarizable S^2−^ compared to O^2−^ can allow for greater flexibility of the local bonding and electronic environment, which may further promote Zn^2+^ mobility.

Based on Rietveld refinement of XRD data (Figures S20d and S21), the delithiated LiTiS_2_ (DLTS) structure exhibits a unit cell parameter of 4.91 Å, substantially larger than that of the DLMTO, which has a unit cell size of 4.05 Å (Figure S21). This confirms that sulfide DRX frameworks provide larger tetrahedral sites compared to oxides.

Electrochemical testing showed that DLTS delivered an initial discharge capacity of ∼182 mAh g^−1^ in Zn‐ion cells, which is comparable to the first charge capacity (∼205 mAh g^−1^) observed in LTS (Figure S20b,c). GITT analysis (Figure S22) further revealed that voltage relaxation during discharge is significantly smaller in the sulfide than in the oxide. Notably, in the following Zn extraction (Figures S20d and S22b), the reversibility was relatively poor, indicating that additional factors beyond Zn^2+^ diffusion kinetics may limit the electrochemical performance. Possible reasons include structural instability of the sulfide framework and electrolyte compatibility issues, which may induce interfacial instability and degrade the reversibility of Zn extraction/insertion.

These results indicate that tailoring the coordination environment through anion substitution can potentially be an effective strategy for facilitating Zn^2+^ mobility in DRX structures, although further study is still needed to improve the reversibility of Zn insertion/extraction. Similar arguments have been proposed to promote Li^+^ diffusion in Li‐based DRX cathodes by expanding the interstitial tetrahedral size [45, 46]. Therefore, designing cathodes with different anion ligands, such as oxysulfides, oxychlorides, and even using selenides, may offer promising opportunities to further enhance Zn diffusion by optimizing anion ionic radius and polarizability.

Importantly, the challenge of high migration barriers is not unique to Zn^2+^ but is a common challenge across multivalent ions. Therefore, our strategy to introduce cation vacancies and mitigate coulombic repulsion may also facilitate diffusion of other multivalent ions within the DRX cathode structures. At present, however, practical implementation for Mg^2+^, Ca^2+^, or Al^3+^ batteries are largely hindered by electrolyte and anode challenges, such as surface passivation on the anode and limited electrochemical stability windows for multivalent electrolytes [1, 7], which constrain compatibility with oxide‐based DRX cathodes. With further progress in electrolyte development, DRX‐based cathodes with modified compositions could potentially represent a viable pathway for advancing a broader range of multivalent battery chemistries.

## 3 Conclusion

To identify new compatible cathode materials for nonaqueous Zn‐ion batteries, DRX structures were investigated given their proven success in Li and Na‐ion systems. Through a combined computational and experimental approach, we demonstrated that introducing cation vacancies is essential for enabling Zn^2+^ diffusion within DRX lattices by substantially reducing the strong electrostatic repulsion encountered during migration. DFT calculations revealed that cation vacancies lower Zn^2+^ migration barriers by nearly a factor of two compared to fully occupied DRX configurations. Experimentally, this design principle was validated using a vacancy‐containing Mn_0.4_Ti_0.4_O_2_ DRX cathode, which delivered a reversible capacity of 170 mAh g^−1^ in a nonaqueous Zn‐ion cell, representing the first successful demonstration of reversible Zn^2+^ (de)intercalation in a DRX oxide. GITT measurements further underscored the intrinsically sluggish nature of Zn transport and the necessity of defect engineering.

In contrast, a broad family of fully occupied Zn‐based DRX materials spanning multiple transition‐metal chemistries exhibited negligible electrochemical activity. This behavior was consistently explained by prohibitively high Zn^2+^ migration barriers, even along nominally favorable 0‐TM pathways, confirming that divalent Zn^2+^ diffusion is locked with close‐packed DRX lattices in the absence of vacancies. Beyond vacancy engineering, we further show that enlarging and softening the anion sublattice, exemplified by sulfide‐based DRX materials, can be a potential strategy for enhancing Zn^2+^ mobility, although further work is still needed to improve the reversibility of Zn insertion/extraction. Together, this work establishes cation vacancy engineering as a key enabler for Zn^2+^ intercalation in DRX cathode and provides fundamental insight into multivalent‐ion transport. These findings not only expand the cathode material landscape for nonaqueous Zn‐ion batteries but also offer general design principles for activating disordered host structures for other multivalent‐ion storage systems.

## Author Contributions

**Zixuan Li**: conceptualization, investigation, writing – original draft, methodology, visualization. **Rui Qi**: investigation, methodology. **Yee Chit Wong**: investigation, methodology. **Longlong Wang**: methodology, investigation. **Jun Chen**: investigation, methodology. **Yi Yuan**: investigation, methodology. **Reinhard J. Maurer**: supervision. **Robert A. House**: validation, writing – review and editing. **Nicholas DM Hine**: supervision, validation, conceptualization. **Peter G. Bruce**: resources, supervision, funding acquisition. **Alex W. Robertson**: resources, supervision, writing – review and editing, validation, funding acquisition.

## Conflicts of Interest

The authors declare no conflicts of interest.

## Supporting information




**Supporting File**: anie73598‐sup‐0001‐SuppMat.docx.

## Data Availability

The data that support the findings of this study are available from the corresponding author upon reasonable request.
