# Supplementary material for: Unlocking Zn‐Ion Diffusion in Disordered Rocksalt Cathodes for Nonaqueous Zn‐Ion Batteries
Source: Angew Chem Int Ed Engl. 2026 Jul 13;65(38):e8007340. doi: 10.1002/anie.8007340 (PMC13573087; doi:10.1002/anie.8007340)
Supplement: Supplementary file 1 [file ANIE-65-e8007340-s001.docx]

**Unlocking Zn-Ion Diffusion in Disordered Rocksalt Cathodes for Nonaqueous Zn-Ion Batteries**

Zixuan Li*^†^,^1^ Rui Qi^†^,^1^ Yee Chit Wong,^2^ Longlong Wang,^1^ Jun Chen,^1^ Yi Yuan,^2^ Reinhard J. Maurer,^2,3,4^ Robert A. House*,^1^ Nicholas DM. Hine*,^2^ Peter G. Bruce*,^1,5^ Alex W. Robertson*^2^

1. Department of Materials, University of Oxford, Parks Road, Oxford, OX1 3PH, UK

2. Department of Physics, University of Warwick, Coventry, CV4 7AL, UK

3. Department of Chemistry, University of Warwick, Coventry, CV4 7AL, UK

4. University of Vienna, Faculty of Physics, 1090, Vienna, Austria

5. Department of Chemistry, University of Oxford, Oxford, OX1 3QZ, UK

^†^ These authors contributed equally

*zixuan.li@materials.ox.ac.uk

*robert.house@materials.ox.ac.uk

*N.D.M.Hine@warwick.ac.uk

*peter.bruce@materials.ox.ac.uk

*alex.w.robertson@warwick.ac.uk

**Experimental Methods**

**Materials Preparation:**

**Synthesis of Zn_x_TM_(2–x)_O_2_:**

Compounds in the Zn_x_TM_(2–x)_O₂ disordered rocksalt (DRX) family including ZnMnO_2_, ZnFeO_2_, ZnCoO_2_, Zn_1.2_Co_0.8_O_2_, ZnNiO_2_, Zn_1.2_Ni_0.8_O_2_, Zn_1.4_Ni_0.6_O_2_, and ZnMg_0.25_Cu_0.25_Co_0.25_Ni_0.25_O_2_, were synthesized via a mechanochemical ball-milling method. ZnO (99%, Sigma-Aldrich) was mixed with transition-metal oxide precursors (MnO, 99%, Sigma-Aldrich; FeO, 99.9%, Sigma-Aldrich; CoO, 99.5%, Sigma-Aldrich; NiO, 99%, Sigma-Aldrich; MgO, 97%, Sigma-Aldrich; CuO, 99% Sigma-Aldrich) in stoichiometric ratios and sealed in air-tight zirconia jars inside an argon-filled glovebox to prevent TM oxidation. For each synthesis, 2 g of precursors were loaded into a 15 mL zirconia jar with 12 g of 5 mm zirconia balls and milled for 8 h at 700 rpm using a Fritsch Pulverisette 7 planetary ball mill. Milling was performed in 5-min intervals with 10-min pauses between cycles to prevent overheating.

**Synthesis of DRX LiMnO_2_:**

DRX LiMnO_2_ was synthesized via a mechanochemical ball-milling method starting from its orthorhombic analogue. Orthorhombic zigzag LiMnO_2_ was synthesized by mixing Li_2_CO_3_ (with 5% excess, 99%, Sigma-Aldrich) and Mn_2_O_3_ (99.9%, Sigma-Aldrich) in a stoichiometric ratio, followed by calcination at 900 °C for 12 h under an Ar atmosphere. DRX LiMnO_2_ was then obtained by loading 2 g of the orthorhombic LiMnO_2_ precursor into a 15 mL zirconia jar with 12 g of 5 mm zirconia balls and milling for 8 h at 600 rpm using a Fritsch Pulverisette 7 planetary ball mill. All sample handling was performed in an Ar-filled glovebox to prevent exposure to moisture and air.

**Synthesis of DRX NaMnO_2_:**

DRX NaMnO_2_ was synthesized via a mechanochemical ball-milling method starting from its layered analogue. Monoclinic layered NaMnO_2_ was synthesized by mixing Na_2_CO_3_ (99.5%, Sigma-Aldrich) and Mn_2_O_3_ (99.9%, Sigma-Aldrich) in a stoichiometric ratio, followed by calcination at 700 °C for 24 h under an Ar atmosphere. DRX NaMnO_2_ was then obtained by loading 2 g of the layered NaMnO_2_ precursor into a 15 mL zirconia jar with 12 g of 5 mm zirconia balls and milling for 8 h at 600 rpm using a Fritsch Pulverisette 7 planetary ball mill. All sample handling was performed in an Ar-filled glovebox to prevent exposure to moisture and air.

**Synthesis of DRX Li_4_Mn_2_O_5_:**

DRX Li_4_Mn_2_O_5_ was synthesized via a two-step ball-milling process. Orthorhombic LiMnO_2_ was mixed with Li_2_O (97%, Sigma-Aldrich) in a 2:1 molar ratio (∼4 g total) and loaded into a 15 mL zirconia jar with 12 g of 5 mm zirconia balls. The mixture was milled for 15 h at 700 rpm using a Fritsch Pulverisette 7 planetary ball mill. Subsequently, 5 wt % acetylene black was added, and the mixture was further milled at 700 rpm for 5 h. All sample handling was performed in an Ar-filled glovebox to prevent exposure to moisture and air.

**Synthesis of DRX Li_1.2_Mn_0.4_Ti_0.2_O_2_:**

DRX Li_1.2_Ti_0.4_Mn_0.4_O_2_ was synthesized by solid-state reaction. Li_2_CO_3_ (with 10% excess, 99%, Sigma-Aldrich), TiO_2_ (99.8%, Sigma-Aldrich), and Mn_2_O_3_ (99.9%, Sigma-Aldrich) precursors were thoroughly mixed in stoichiometric proportions and calcined at 950 °C for 12 h under an Ar atmosphere. To optimize electrochemical performance, the as-synthesized Li_1.2_Ti_0.4_Mn_0.4_O_2_ (~2 g) was further pulverized by ball milling. The material was loaded into a 15 mL zirconia jar with 12 g of 5 mm zirconia balls and milled for 8 h at either 300 rpm or 600 rpm using a Fritsch Pulverisette 7 planetary ball mill, producing samples with different degrees of pulverization.

**Synthesis of DRX LiTiS_2_:**

DRX LiTiS_2_ was synthesized via a mechanochemical ball-milling method starting from its layered analogue. Layered LiTiS_2_ was synthesized via solid-state reaction using stoichiometric amounts of Li_2_S (99.98%, Sigma-Aldrich), Ti (99.5%, Thermo Scientific) and elemental S (99.98%, Sigma-Aldrich). The precursor powders were thoroughly mixed by hand grinding and pelletized in an Ar-filled glove box. The resulting pellets were loaded into graphite crucibles and placed inside quartz tubes, which were then sealed under vacuum (~10^−5^ mbar). The sealed tubes were slowly heated to 750^o^C over 25 hours, held for 36 hours, and then quenched in air. All sample handling was performed in an Ar-filled glovebox to prevent exposure to moisture and air.

**Trial of Zn_0.5_MnO₂ and Zn_0.75_FeO₂:**

Zn_0.5_MnO_2_ DRX was attempted by ball milling ZnO, MnO, and MnO_2_ (99%, Sigma-Aldrich) in a 1:1:1 molar ratio at 700 rpm for 8 h. Zn_0.75_FeO_2_ DRX was attempted by ball milling ZnO, FeO, and Fe_2_O_3_ (96%, Sigma-Aldrich) in a 3:2:1 molar ratio at 700 rpm for 8 h. However, the resulting products were not phase-pure.

**Electrochemistry:**

Free-standing cathodes, including Zn_x_TM_(2–x)_O_2_, LiMnO_2_, NaMnO_2_, Li_1.2_Ti_0.4_Mn_0.4_O_2_, and LiTiS_2_ films, were prepared by mixing the active material, acetylene black, and PTFE in a 7:2:1 mass ratio using a mortar and pestle. For Li_4_Mn_2_O_5_, which already contained 5 wt % carbon from synthesis, the mixing ratio was adjusted to 7.37 parts (as-synthesized Li_4_Mn_2_O_5_):1.63 parts additional carbon:1 part PTFE, ensuring the final composite maintained the standard 7:2:1 mass ratio of active material, carbon, and PTFE.

For Zn ion batteries (ZIBs), Zn metal foil was used as the anode with 1 M Zn(TFSI)_2_ in acetonitrile as the electrolyte. For Li cells, Li metal discs served as the anode with 1 M LiPF_6_ in a 1:1 (v/v) mixture of ethylene carbonate and dimethyl carbonate (EC:DMC, Aldrich) as the electrolyte. For Na cells, Na metal discs (pressed onto Al foil) were used as the anode with 1 M NaPF_6_ in a 1:1 (v/v) mixture of EC:DMC (E-lyte Innovations) as the electrolyte. Electrode preparation and cell assembly were performed entirely in an Ar-filled glovebox. Electrochemical testing was conducted on a Maccor Series 4000 system at room temperature. Electrochemical testing, including galvanostatic cycling, was performed on a Maccor Series 4000 system at room temperature, while galvanostatic intermittent titration technique (GITT) measurements were carried out using a BioLogic VMP3.

**Characterization:**

The ex-situ characterization was conducted on cathode film or powder samples after charging/discharging to specific states of charge and disassembly from coin cells. The cathode film or powder was thoroughly rinsed several times with acetonitrile or DMC to remove any residual electrolyte, followed by vacuum drying.

Powder X-ray diffraction (PXRD) was performed using a Rigaku Miniflex diffractometer with Cu Kα1 radiation. The PXRD data were analyzed using the Rietveld refinement method with GSAS-II software.

Scanning electron microscopy (SEM) images were obtained using a Carl Zeiss Merlin field emission scanning electron microscope. High resolution transmission electron microscopy (HRTEM) was carried out using a JEOL JEM-2100 operating at 200 kV. Scanning transmission electron microscopy (STEM) with high-angle annular dark-field (HAADF) imaging and energy-dispersive X-ray spectroscopy (EDX) were performed using an aberration-corrected JEOL ARM 200F, equipped with an Oxford Instruments windowless EDX detector.

X-ray absorption near-edge structure (XANES) was conducted at beamline B18 of the Diamond synchrotron source and was performed in transmission and fluorescence yield. Soft X-ray Absorption Spectra (sXAS) were conducted at beamline WERA at the KIT Light Source in Germany.

Operando differential electrochemical mass spectrometry (DEMS) analysis was performed to study the O_2_ evolution during cell cycling. The set up consisted of a quadrupole mass spectrometer (Thermos Fischer) equipped with a turbomolecular pump (Pfeiffer Vacuum) and mass-flow controllers (Bronkhorst). Two electrode type cells (ECC-Std from EL-CELL) with gas inlet and outlet ports were used for the operando measurements. The cell consisted of a Zn anode, 1 M Zn(TFSI)_2_ in PC electrolyte and a cathode film as described above.

**DFT Calculation:**

The electronic structure calculation software CASTEP (version 24.1^1^) was used to study the DRX materials. Since nudged elastic band (NEB) calculations are computationally expensive, a relatively small supercell of size 2×2×2 was simulated, containing a total of 64 atoms. The calculated lattice parameters for the 2×2×2 ZnMnO_2_ and LiMnO_2_ are 8.89 Å and 8.45 Å respectively. Convergence tests on forces and energies were performed on pristine and vacancy-containing structures ranging from the primitive unit cell to 3×3×3 supercells.

A relatively coarse k-point sampling of 3×3×3 on the supercell was used based on the convergence tests, noting that the fully disordered structures exhibit strong anisotropy and low symmetry so the available reduction of k-point grids due to symmetry is minimal. Since both LiMnO_2_ and ZnMnO_2_ are correlated oxides with localized *d* electrons, it was necessary to apply a Hubbard U correction^2^ for Mn to obtain reliable density of states and band gaps, ensuring that the materials are not erroneously predicted as metallic. The Hubbard U parameter was determined through analysis of density of states on the spinel like phase of LiMnO_2_ and ZnMnO_2_ when increasing the value of U, and our final value for U (4.0 eV) is also similar to values reported in previous studies.^3,4^

The generalized gradient approximation PBE functional^5^ was chosen for our calculations. A plane wave cutoff energy of 1000 eV was used, which provided sufficient convergence. On-the-fly ultrasoft pseudopotentials (C19) as implemented in CASTEP were used. Climbing image NEB (CI-NEB) calculations^6,7^ were performed using 11 images. The NEB optimizations calculations were performed using the Atomic Simulation Environment software (ASE),^8^ utilizing CASTEP calculators. The images were initially generated by interpolation using the image dependent pair potential (IDPP) method,^9^ and the maximum force component on all images was converged to 0.05 eV Å^-1^ during the NEB calculation.

Since monoclinic layered LiMnO_2_ is often regarded as a magnetic material,^10,11^ it is essential to include spin polarization in our calculations. We achieve this by assigning initial spin moments to the transition metal ions, giving positive spin moments of 4.0 or 5.0 to the Mn ions in LiMnO_2_ and ZnMnO_2_ respectively. Both antiferromagnetic (AFM) and ferromagnetic (FM) configurations were investigated during the preliminary tests, and the individual atomic spin moments are allowed to change during relaxation and NEB calculations. Due to the complex spin energy landscape, the choice of initial spin configuration can influence the system relaxation toward a state resembling the starting configuration even if it is not the global minimum energy spin state. For DRX, the possible spin configurations are even less enumerable due to potential local short-range ordering, and either FM or AFM configurations could be energetically favorable depending on the specific atomic arrangement. To simplify the NEB calculations, we restrict ourselves to only consider FM configurations in this study.

Due to the disordered arrangement of cations in DRX, numerous possible ion migration configurations exist. Only one specific case is calculated which is shown in Figure S6. Although annealing could, in principle, be used to discover lower-energy configurations, this would be computationally prohibitive here given the vast number of possible cation arrangements. While these DFT calculations provide valuable insight into the fundamental transport behavior of Zn^2+^ and Li^+^ in different migration channels, they represent only a single local configuration. A comprehensive analysis of the distribution of barrier heights as a function of cation disorder would require extensive sampling over local environments.

**Table S1.** Comparison of the electrochemical performance of DLMTO with that of reported cathode materials for non-aqueous zinc-ion batteries.

| Cathodes | Capacity (mAh g^-1^) | Voltage range (V) | Cycle performance | Ref |
| --- | --- | --- | --- | --- |
| Mn_0.4_Ti_0.4_O_2_ (DLMTO) | 170 | 0.2-2.3 | 51.2% after 60 cycles | This work |
| δ‑MnO_2_ | 123 | 0.05-1.9 | 60% after 124 cycles | 12 |
| ZnNi_1/2_Mn_1/2_CoO_4_ | 180 | 0.8-2.15 | 81.6% after 200 cycles | 13 |
| K_1.6_Mn_1.2_Fe(CN)_6_ | 66 | 0.7-1.9 | 94% after 8500 cycles | 14 |
| Na_3_V_2_(PO_4_)_2_F_3_ | 49 | 0.8-1.8 | 22.4% after 10 cycles | 15 |
| γ′-V_2_O_5_ | 130 | 0.3-2 | 90% after 100 cycles | 16 |
| CoS_2_ | 139 | 0.05-1.6 | 83% after 100 cycles | 17 |
| VS_2_ | 153.7 | 0.3-1 | 95.7 after 500 cycles | 18 |

**Figure S1.** The SEM of as-synthesized ZnMnO_2_.

**Figure S2.** (a) Galvanostatic load curve of ZnMnO_2_ ball-milled with carbon (7:2 mass ratio) at 300 rpm for 6 h, tested in organic electrolyte at 10 mA g^-1^. (b) Ex situ Mn K-edge XANES spectra of ZnMnO_2_ at P, FC, and FD states.

**Figure S3.** (a) Rietveld refinement of XRD data for ZnFeO_2_ with corresponding unit cell parameters. (b) Galvanostatic load curve of ZnFeO_2_ in organic electrolyte at 10 mA g^-1^. (c) Ex situ Fe K-edge XANES spectra of ZnFeO_2_ at P, FC, and FD states. (d) Rietveld refinement of XRD data for ZnCoO_2_ with corresponding unit cell parameters. (e) Galvanostatic load curve of ZnCoO_2_ in organic electrolyte at 10 mA g^-1^. (f) Rietveld refinement of XRD data for ZnNiO_2_ with corresponding unit cell parameters. (g) Galvanostatic load curve of ZnNiO_2_ in organic electrolyte at 10 mA g^-1^. (h) Ex situ Ni K-edge XANES spectra of ZnNiO_2_ at P, FC, and FD states.

**Figure S4.** (a) Rietveld refinement of XRD data for ZnMg_0.25_Cu_0.25_Co_0.25_Ni_0.25_O_2_ with corresponding unit cell parameters. (b) Galvanostatic load curve of ZnMg_0.25_Cu_0.25_Co_0.25_Ni_0.25_O_2_ in organic electrolyte at 10 mA g^-1^.

**Figure S5.** (a) Rietveld refinement of XRD pattern for Zn_1.2_Co_0.8_O_2_ with corresponding unit cell parameters. (b) Galvanostatic cycling curve of Zn_1.2_Co_0.8_O_2_ in organic electrolyte at 10 mA g^-1^. (c) Rietveld refinement of XRD pattern for Zn_1.2_Ni_0.8_O_2_ with corresponding unit cell parameters. (d) Galvanostatic cycling curve of Zn_1.2_Ni_0.8_O_2_ in organic electrolyte at 10 mA g^-1^. (e) Rietveld refinement of XRD pattern for Zn_1.4_Ni_0.6_O_2_ with corresponding unit cell parameters. (f) Galvanostatic cycling curve of Zn_1.4_Ni_0.6_O_2_ in organic electrolyte at 10 mA g^-1^.

**Figure S6.** Selected representative ion migration pathways for 0-TM, 1-TM, and 2-TM channels.

**Figure S7.** XRD patterns of as-synthesised target compounds Zn_0.5_MnO_2_ and Zn_0.75_FeO_2_ obtained by mechanochemical ball milling. The impurity comes from unreacted ZnO.

**Figure S8.** (a) Charge curve of LiMnO_2_ DRX in a Li-ion battery at 10 mA g^-1^. (b) Ex situ powder XRD of LiMnO_2_ at different states of charge, corresponding to the points marked in panel (a). (c) Weight fractions of spinel and DRX phases at the corresponding states, obtained from Rietveld refinement of the XRD data.

**Figure S9.** Rietveld refinement of XRD patterns for pristine LMO, DLMO, DLMO ZnFD, and DLMO ZnFC. LMO represents LiMnO_2_, DLMO represents delithiated LiMnO_2_, DLMO ZnFD represents the full discharge state, and DLMO ZnFC represents the full charge state.

**Figure S10.** SEM images of as-synthesised (a) LiMnO_2_, (b) Li_4_Mn_2_O_5_ and (c) NaMnO_2_.

**Figure S11.** (a) Charge curve of Li_4_Mn_2_O_5_ (LMO425) DRX in a Li-ion battery at 10 mA g^-1^. (b) Discharge/charge profile of delithiated Li_4_Mn_2_O_5_ (DLMO425) in a Zn-ion battery, with inset showing cycling stability at 10 mA g⁻¹. (c) Ex situ powder XRD of pristine LMO425, DLMO425, DLMO425 ZnFD, and DLMO425 ZnFC. (d) Ex situ Mn K-edge XANES spectra of LMO425, DLMO425, DLMO425 ZnFD, and DLMO425 ZnFC. LMO425 represents Li_4_Mn_2_O_5_, DLMO425 represents delithiated Li_4_Mn_2_O_5_, DLMO425 ZnFD represents the full discharge state in ZIBs, and DLMO425 ZnFC represents the full charge state in ZIBs.

**Figure S12.** Rietveld refinement of XRD patterns for LMO425, DLMO425, DLMO425 ZnFD, and DLMO425 ZnFC.

**Figure S13.** (a) Charge curve of NaMnO_2_ (NMO) DRX in a Na-ion battery at 10 mA g⁻¹. (b) Discharge/charge profile of desodiated NaMnO_2_ (DNMO) in a Zn-ion battery, with inset showing cycling stability at 10 mA g⁻¹. (c) Ex situ powder XRD of pristine NMO, DNMO, DNMO ZnFD, and DNMO ZnFC. (c) Ex situ Mn K-edge XANES spectra of NMO, DNMO, DNMO ZnFD, and DNMO ZnFC. NMO represents NaMnO_2_, DNMO represents desodiated NaMnO_2_, DNMO ZnFD represents the full discharge state in ZIBs, and DNMO ZnFC represents the full charge state in ZIBs.

**Figure S14.** Rietveld refinement of XRD patterns for NMO, DNMO, DNMO ZnFD, and DNMO ZnFC.

**Figure S15.** SEM images of Li_1.2_Mn_0.4_Ti_0.4_O_2_: (a) as-synthesised by solid-state method, (b) after ball milling at 300 rpm for 6 h, and (c) after ball milling at 600 rpm for 6 h.

**Figure S16.** Rietveld refinement of XRD patterns for Li_1.2_Mn_0.4_Ti_0.4_O_2_ (LMTO, ball-milled at 600 rpm for 6 h), DLMTO, DLMTO ZnFD, and DLMTO ZnFC. LMTO represents Li_1.2_Mn_0.4_Ti_0.4_O_2_, DLMTO represents delithiated Li_1.2_Mn_0.4_Ti_0.4_O_2_, DLMTO ZnFD represents the full discharge state in ZIBs, and DLMTO ZnFC represents the full charge state in ZIBs.

**Figure S17.** Differential electrochemical mass spectrometry (DEMS) profiles for O_2_ gas evolution of DLMTO in ZIB at a current rate of 20 mA g^-1^.

**Figure S18.** (a) Charge curve of Li_1.2_Mn_0.4_Ti_0.4_O_2_ DRX (LMTO, ball-milled at 300 rpm for 6 h) in a Li-ion battery at 10 mA g^-1^. (b) Discharge/charge profile of delithiated Li_1.2_Mn_0.4_Ti_0.4_O_2_ (DLMTO) in a Zn-ion battery, with inset showing cycling stability at 10 mA g⁻¹. (c) Ex situ powder XRD of LMTO, DLMTO, DLMTO ZnFD, and DLMTO ZnFC.

**Figure S19.** GITT profiles of DRX DLMTO (ball-milled at 600 rpm for 6 h) measured at 20 mA g^-1^ with 10 min current pulses followed by 1 h relaxation to reach equilibrium, tested in (a) a Zn-ion battery and (b) a Li-ion battery. The change of voltage after relaxation (ΔV) is plotted against the capacity before relaxation. The orange data represent the discharge process, while the green data represent the charge process.

**Figure S20.** (a) Schematic of sulphide DRX, highlighting its larger tetrahedral height compared to oxide DRX. (b) Charge curve of LiTiS_2_ (LTS) DRX in a Li-ion battery at 10 mA g^-1^. (c) Discharge/charge profile of delithiated LiTiS_2_ (DLTS) in a Zn-ion battery at 10 mA g^-1^. (d) Ex situ powder XRD and (e) ex situ Ti K-edge XANES spectra of LTS, DLTS, DLTS ZnFD, and DLTS ZnFC. LTS represents LiTiS_2_, DLTS represents delithiated LiTiS_2_, DLTS ZnFD represents the full discharge state in ZIBs, and DLTS ZnFC represents the full charge state in ZIBs.

**Figure S21.** Rietveld refinement of XRD patterns LTS, DLTS, DLTS ZnFD, and DLTS ZnFC.

**Figure S22.** GITT profile of (a) DLMTO DRX (ball-milled at 600 rpm for 6 h) and (b) DLTS measured at 10 mA g^-1^ with 1h current pulses followed by 4 h relaxation, tested in a Zn-ion battery. The change of voltage after relaxation (ΔV) is plotted against the capacity before relaxation. The orange data represent the discharge process, while the green data represent the charge process.

**Reference**

1	Clark, S. J. *et al.* First principles methods using CASTEP. *Zeitschrift für kristallographie*, *Cryst. Mater.* **220**, 567-570 (2005).

2	Dudarev, S. L., Botton, G. A., Savrasov, S. Y., Humphreys, C. & Sutton, A. P. Electron-energy-loss spectra and the structural stability of nickel oxide: An LSDA+ U study. *Phys. Rev. B* **57**, 1505–1509 (1998).

3	Tsebesebe, N., Kgatwane, K., Ledwaba, R. & Ngoepe, P. Investigating the Structural and Electronic Properties of LiMO_2_ (M: Mn, Ni, Co) as Potential Cathode Materials: A DFT Study. *J. Phys. Conf. Ser.* **2298**, 012010 (2022).

4	Pulido, R. *et al.* Experimental and density functional theory study of the Li^+^ desorption in spinel/layered lithium manganese oxide nanocomposites using HCl. *Chem. Eng. J.* **441**, 136019 (2022).

5	Perdew, J. P., Burke, K. & Ernzerhof, M. Generalized gradient approximation made simple. *Physical review letters* **77**, 3865 (1996).

6	Jónsson, H., Mills, G. & Jacobsen, K. W. Nudged elastic band method for finding minimum energy paths of transitions. in *Classical and Quantum Dynamics in Condensed Phase Simulations* 385–404 (WORLD SCIENTIFIC, 1998). doi:10.1142/9789812839664_0016.

7	Henkelman, G., Uberuaga, B. P. & Jónsson, H. A climbing image nudged elastic band method for finding saddle points and minimum energy paths. *J. Chem. Phys.* **113**, 9901–9904 (2000).

8	Hjorth Larsen, A. *et al.* The atomic simulation environment—a Python library for working with atoms. *J. Phys. Condens. Matter* **29**, 273002 (2017).

9	Smidstrup, S., Pedersen, A., Stokbro, K. & Jónsson, H. Improved initial guess for minimum energy path calculations. *J. Chem. Phys.* **140**, 214106 (2014).

10	Singh, D. J. Magnetic and electronic properties of LiMnO_2_s. *Phys. Rev. B* **55**, 309–312 (1997).

11	Mackrodt, W. C. & Williamson, E.-A. First-principles Hartree-Fock description of the electronic structure of monoclinic C 2/m Li_x_MnO_2_ (1≥ x≥ 0). *Philos. Mag. B* **77**, 1077–1092 (1998).

12	Han, S.-D. *et al.* Mechanism of Zn insertion into nanostructured δ-MnO2: a nonaqueous rechargeable Zn metal battery. *Chemistry of Materials* **29**, 4874–4884 (2017).

13	Pan, C., Zhang, R., Nuzzo, R. G. & Gewirth, A. A. ZnNi_x_Mn_x_Co_2–2x_O_4_ spinel as a high‐voltage and high‐capacity cathode material for nonaqueous Zn‐ion batteries. *Adv. Energy Mater.* **8**, 1800589 (2018).

14	Li, Q., Ma, K., Yang, G. & Wang, C. High-voltage non-aqueous Zn/K_1.6_Mn_1.2_Fe(CN)_6_ batteries with zero capacity loss in extremely long working duration. *Energy Storage Mater.* **29**, 246–253 (2020).

15	Park, M. J. & Manthiram, A. Unveiling the charge storage mechanism in nonaqueous and aqueous Zn/Na_3_V_2_(PO_4_)_2_F_3_ batteries. *ACS Appl. Energy Mater.* **3**, 5015–5023 (2020). 16	Bhatia, A., Xu, J., Pereira-Ramos, J.-P., Rousse, G. & Baddour-Hadjean, R. γ′-V_2_O_5_ polymorph: a genuine Zn intercalation material for nonaqueous rechargeable batteries. *Chem. Mater.* **34**, 1203–1212 (2021).

17	Zhang, R., Pan, C., Nuzzo, R. G. & Gewirth, A. A. CoS_2_ as a Sulfur Redox-Active Cathode Material for High-Capacity Nonaqueous Zn Batteries. *J. Phys. Chem. C* **123**, 8740–8745 (2019).

18	Naveed, A. *et al.* A highly reversible Zn anode with intrinsically safe organic electrolyte for long‐cycle‐life batteries. *Adv. Mater.* **31**, 1900668 (2019).
